# Antiviral susceptibility of clade 2.3.4.4b highly pathogenic avian influenza A(H5N1) viruses isolated from birds and mammals in the United States, 2022

**DOI:** 10.1016/j.antiviral.2023.105679

**Published:** 2023-07-24

**Authors:** Ha T. Nguyen, Anton Chesnokov, Juan De La Cruz, Philippe Noriel Q. Pascua, Vasiliy P. Mishin, Yunho Jang, Joyce Jones, Han Di, Andrei A. Ivashchenko, Mary Lea Killian, Mia K. Torchetti, Kristina Lantz, David E. Wentworth, Charles T. Davis, Alexandre V. Ivachtchenko, Larisa V. Gubareva

**Affiliations:** a Influenza Division, National Center for Immunization and Respiratory Diseases, Centers for Disease Control and Prevention (CDC), Atlanta, GA, USA; b ChemDiv, 12760 High Bluff Drive, Ste. 370, San Diego, CA 92130, USA; c National Veterinary Services Laboratories, U.S. Department of Agriculture (USDA), Ames, IA 50010, USA; d AVISA LLC, 1835 E. Hallandale Beach Blvd, #442, Hallandale Beach, FL 33009, USA

**Keywords:** Neuraminidase inhibitors, CEN inhibitor, Baloxavir, AV5080, Antiviral resistance

## Abstract

Clade 2.3.4.4b highly pathogenic avian influenza (HPAI) A(H5N1) viruses that are responsible for devastating outbreaks in birds and mammals pose a potential threat to public health. Here, we evaluated their susceptibility to influenza antivirals. Of 1,015 sequences of HPAI A(H5N1) viruses collected in the United States during 2022, eight viruses (~0.8%) had a molecular marker of drug resistance to an FDA-approved antiviral: three adamantane-resistant (M2-V27A), four oseltamivir-resistant (NA-H275Y), and one baloxavir-resistant (PA-I38T). Additionally, 31 viruses contained mutations that may reduce susceptibility to inhibitors of neuraminidase (NA) (n = 20) or cap-dependent endonuclease (CEN) (n = 11). A panel of 22 representative viruses was tested phenotypically. Overall, clade 2.3.4.4b A(H5N1) viruses lacking recognized resistance mutations were susceptible to FDA-approved antivirals. Oseltamivir was least potent at inhibiting NA activity, while the investigational NA inhibitor AV5080 was most potent, including against NA mutants. A novel NA substitution T438N conferred 12-fold reduced inhibition by zanamivir, and in combination with the known marker N295S, synergistically affected susceptibility to all five NA inhibitors. In cell culture-based assays HINT and IRINA, the PA-I38T virus displayed 75- to 108-fold and 37- to 78-fold reduced susceptibility to CEN inhibitors, baloxavir and the investigational AV5116, respectively. Viruses with PA-I38M or PA-A37T showed 5- to 10-fold reduced susceptibilities. As HPAI A(H5N1) viruses continue to circulate and evolve, close monitoring of drug susceptibility is needed for risk assessment and to inform decisions regarding antiviral stockpiling.

Highly pathogenic avian influenza (HPAI) A(H5N1) viruses persist in wild birds in many countries and cause deadly outbreaks in poultry and mammals worldwide. They are also known to cause sporadic human infections of various severity. Their circulation in wild birds is of public health concern as they may acquire the ability to effectively infect humans and transmit person-to-person (Krammer and Schultz-Cherry, 2023). HPAI A(H5N1) viruses display remarkable genetic heterogeneity due to genetic drift and reassortment that has resulted in multiple phylogenetically distinct hemagglutinin clades and various genotypes. In December 2021, clade 2.3.4.4b HPAI A(H5N1) viruses of Eurasian-origin were first detected on the Atlantic Coasts of North America (Alkie et al., 2023; Bevins et al., 2022). These viruses then underwent reassortment where they exchanged genes with those from North American lineage low pathogenic avian influenza viruses (Alkie et al., 2023; Elsmo et al., 2023; Kandeil et al., 2023). By the end of 2022, clade 2.3.4.4b HPAI A(H5N1) viruses were found in wild birds, domestic poultry, or wild mammals in nearly all continental states of the United States (US) (CDC, 2023).

Vaccines and antivirals are used to control seasonal influenza infections and are key components of pandemic preparedness. Antivirals can mitigate the impact of outbreaks in humans caused by zoonotic viruses for which vaccines may not be readily available. However, influenza viruses can become less susceptible to antivirals by acquiring genomic changes due to spontaneous mutations, gene reassortment, or selective pressure from antiviral treatment. To manage human influenza A virus infections, Food and Drug Administration (FDA) has approved antivirals from three classes: M2 blockers (adamantanes), neuraminidase (NA) inhibitors, and the cap-dependent endonuclease (CEN) inhibitor of the polymerase acid (PA) protein (Jones et al., 2023). Due to resistance of seasonal influenza A viruses to M2 blockers, Centers for Disease Control and Prevention (CDC) recommends the use of NA inhibitors oseltamivir, zanamivir, and peramivir and a CEN inhibitor, baloxavir (CDC, 2022). Laninamivir is another NA inhibitor which is approved in Japan (Ikematsu and Kawai, 2011).

Evaluation of antiviral susceptibility is an integral part of the risk assessment conducted on influenza viruses that pose a threat to public health (Cox et al., 2014; Global Influenza Programme WEP, 2020). In this study, clade 2.3.4.4b HPAI A(H5N1) viruses collected from birds and mammals in the US during 2022 were assessed for their susceptibility to FDA-approved and two investigational antivirals. First, virus genome sequences (n = 1,015) obtained from the Global Initiative on Sharing All Influenza Data (GISAID) database were examined for known markers of resistance with perceived clinical relevance. Of the known markers of adamantane resistance (Gubareva and Hayden, 2006), M2-V27A was detected in three viruses collected from domestic birds in South Dakota on the same day (Table S1). Four viruses collected from birds in New England states had NA-H275Y, the common marker of oseltamivir resistance which may also confer resistance to peramivir (Table S1) (Lee and Hurt, 2018). Furthermore, a virus from a chicken contained PA-I38T, the main determinant for baloxavir resistance (Gubareva et al., 2019). Hence, viruses containing common markers of resistance to any FDA-approved antivirals (M2-V27A, NA-H275Y, PA-I38T) were detected at a low frequency (0.8%, 8 of 1015). Additionally, sequences were also analyzed for other amino acid substitutions previously reported to confer reduced inhibition by NA inhibitor(s) in NA inhibition (NI) assays or reduced baloxavir susceptibility in cell culture-based assays (WHO, 2023). Two viruses, from a chicken and a skunk, containing NA-I117T were detected in different states. In Florida, a virus collected from a dolphin had NA-S247N, while two viruses from vultures had NA-N295S (Table S1). We also flagged viruses that contained NA sequence changes with unknown effects on drug susceptibility because these occurred at residues previously implicated in reduced inhibition. For example, an NA-V149A induced 8-fold reduced inhibition by zanamivir in a clade 1 A(H5N1) virus (Naughtin et al., 2011). Therefore, we flagged viruses with V149F (n = 2) and V149I (n = 8). Similarly, the following NA substitutions were flagged: Q136P (n = 1), D199E (n = 1), S247G (n = 1), and N295D (n = 1) (Table S1). For substitutions in PA, we flagged viruses with changes previously reported to confer reduced baloxavir susceptibility in seasonal influenza viruses: K34R (n = 1), A36V (n = 2), A37T (n = 4), I38M (n = 1), and I38V (n = 3) (Table S1) (WHO, 2023).

To conduct phenotypic testing, we assembled a panel of clade 2.3.4.4b A(H5N1) viruses (n = 22) that were collected from different animal species and different genotypes (Elsmo et al., 2023), including viruses with flagged mutations that were available for testing (Table S2). A/American wigeon/South Carolina/2021, collected at the beginning of the US outbreak, was also included. Viruses were tested in a fluorescence-based NI assay (Okomo-Adhiambo et al., 2010) with four marketed NA inhibitors and AV5080, an oral NA inhibitor currently undergoing clinical trials (Ivachtchenko et al., 2014; Jones et al., 2023). The baseline susceptibility was determined by calculating the median IC_50_ (50% inhibitory concentration) of clade 2.3.4.4b viruses lacking any of the known or suspected NA markers of reduced inhibition (n = 15, Table 1). The median IC_50_s were in a sub-nanomolar range for all NA inhibitors, except for oseltamivir, which was 13- to 27-fold less effective than other marketed NA inhibitors. Notably, AV5080 was the most potent at inhibiting the NA activity of clade 2.3.4.4b A(H5N1) viruses, especially compared to oseltamivir (67-fold). Next, we evaluated the effect of each NA mutation on drug susceptibility by comparing the IC_50_ to the determined baseline. Kode et al. (2019) reported that NA-I117T in a clade 2.2 A(H5N1) virus conferred 12- to 19-fold reduced inhibition by oseltamivir and zanamivir. However, we did not observe any effect on susceptibility to NA inhibitors, whether comparing to the baseline or IC_50_ of NA sequence-matched wildtype virus (Table S2). This apparent discrepancy may point to a clade-specific effect or differences in calculating fold-change. NA-S247N reportedly reduced oseltamivir inhibition by 24-fold in a clade 2.3.4 virus (Boltz et al., 2010), whereas in a clade 2.3.4.4b virus tested here, NA-S247N only conferred 4- to 6-fold reduction by oseltamivir and peramivir. NA-V149I was found in a clade 7.1 A(H5N1) virus but was not evaluated for its effect on drug susceptibility (Creanga et al., 2017). Here, NA-V149I did not alter susceptibility to NA inhibitors. A previously unreported NA-N295D mutation conferred 4-fold reduced inhibition by zanamivir and AV5080. In different clades of A (H5N1) viruses, NA-N295S was reported to confer 12- to 126-fold reduced inhibition by oseltamivir (Earhart et al., 2009; Le et al., 2005; McKimm-Breschkin et al., 2018; Sood et al., 2018). The two A(H5N1) viruses collected from vultures in Florida that had NA-N295S additionally contained a threonine-to-asparagine substitution at residue 438 (Table 1), which is highly conserved among influenza A viruses (Yasuhara et al., 2022). Notably, this dual N295S+T438N mutation was detected in the original specimens and their respective isolates. However, sequence analysis showed the presence of mixed virus populations at both residues in one of the bird viruses, A/black vulture/Florida/22-012333-001/2022 (Table 1). This isolate was subjected to conventional limiting dilution procedure in MDCK-SIAT1 cells to separate NA variant, yielding three distinct virus clones containing N295S, T438N, or both N295S+T438N (Table 1). By itself, NA-N295S conferred 14-fold reduced inhibition by oseltamivir and 3- to 7-fold reduced inhibition by the other NA inhibitors, whereas NA-T438N conferred ~12-fold reduced inhibition by zanamivir only. The effect of the dual NA mutation N295S+T438N was synergistic, causing 16- to 76-fold reduced inhibition by all NA inhibitors; this is similar to the inhibition profile of the virus with no detected mixture (Table 1).

The A(H5N1) viruses were then tested for their susceptibility to the CEN inhibitors baloxavir (acid) and AV5116 (Ivashchenko et al., 2021) using cell culture-based assays HINT (high-content imaging neutralization test) (Gubareva et al., 2019) and IRINA (influenza replication inhibition neuraminidase-based assay) (Patel et al., 2022). To minimize the effect of different growth kinetics between viruses, the time of virus replication was limited to 8–10 h (Supp. Fig. 1). The baseline susceptibilities to CEN inhibitors were determined by calculating the median EC_50_ (50% effective concentration) for viruses (n = 17) that lack flagged PA mutations (Table 2). The median EC_50_s fell into a sub-nanomolar range for both baloxavir and AV5116. Overall, clade 2.3.4.4b A (H5N1) viruses seem to be slightly (1.2- to 5-fold) more susceptible to these antivirals than the reference human influenza A viruses. EC_50_s determined by HINT tended to be higher compared to those determined by IRINA, but this difference was small (~2-fold) (Table 2 and S2). The clade 2.3.4.4b A(H5N1) virus with PA-I38T displayed 75- to 108-fold reduced susceptibility to baloxavir and 37- to 78-fold reduced susceptibility to AV5116. These results were similar to those obtained for the reference baloxavir-resistant A(H1N1)pdm09 virus with PA-I38T (Table 2). Expectedly, the effect of PA-I38M was less pronounced. This change caused 6- to 10-fold reduction in baloxavir and AV5116 susceptibility, which is similar to the human A(H3N2) virus with PA-I38M. Two available viruses with PA-A37T showed 5- to 6-fold reduced susceptibility to the CEN inhibitors.

Sequence-based analysis provides a high-throughput means to monitor susceptibility of emerging and circulating viruses and is the primary tool in virological surveillance. This approach is especially valuable for HPAI viruses as sequencing does not require enhanced biosafety containment and can be carried out on original specimens using complete genome PCR primers universal to all influenza A virus subtypes. However, sequence-based assessment has its limitations, particularly with respect to susceptibility to newer antivirals with limited or no phenotypic data. It is also well-recognized that effects of amino acid substitutions can be subtype- and clade-specific. Thus, phenotypic testing serves two main purposes: to establish baseline susceptibility for newly emerged viruses and to assess the effect of identified mutations on drug phenotype. We and others (Kandeil et al., 2023) showed that clade 2.3.4.4b viruses collected in the US in 2021–2022 were susceptible to NA and CEN inhibitors. Our study demonstrated that the oseltamivir susceptibility baseline for clade 2.3.4.4b viruses was somewhat higher compared to human A(H1N1) pdm09 viruses. Similar observations were made in the past for certain genetic clades of A(H5N1) viruses (Creanga et al., 2017; Le et al., 2008; McKimm-Breschkin et al., 2007, 2018; Nguyen et al., 2013). Here, we also report the detection of sporadic mutations in clade 2.3.4.4b viruses that may confer resistance or reduced susceptibility to FDA-approved antivirals. The four identified oseltamivir-resistant viruses with NA-H275Y were not available for phenotypic testing, however, they are expected to display highly reduced inhibition in NI assays (>100-fold) (Lee and Hurt, 2018). Notably, two viruses with the dual NA mutation N295S+T438N displayed oseltamivir IC_50_s similar to that of the human A(H1N1)pdm09 virus with NA-H275Y. Not only did the dual mutation confer reduced inhibition by all NA inhibitors, but it also appeared to have lowered the NA activity of these viruses (Table S4) which may reduce virus fitness in mucin-rich environment, such as the upper respiratory tract of humans. Because these dual-mutant viruses were collected from vultures on the same day in Florida, it is possible that they acquired these drug-resistant viruses from the same source (e.g., consuming the same carcass of an infected animal). However, bird-to-bird transmission also cannot be ruled out. Notably, limited transmission of oseltamivir-resistant viruses with NA-H275Y was recently reported in birds (Skog et al., 2023). Clusters of drug-resistant viruses can serve as indicators of local transmission. The four oseltamivir-resistant viruses with NA-H275Y identified in this study did not form a cluster as they were collected in different places and at different times. The detection of a cluster of adamantane-resistant viruses, however, is not surprising as such viruses are known to transmit well (Gubareva and Hayden, 2006). We also detected a small cluster of three viruses with PA-A37T that displayed reduced baloxavir susceptibility. To the best of our knowledge, this is the first report of a potential local transmission of such viruses among domestic poultry. To improve risk assessment, studies are needed to evaluate the replicative fitness of A(H5N1) viruses with PA-A37T and other PA mutations affecting susceptibility to CEN inhibitors. Compared to the currently marketed antivirals, we also showed that the investigational drugs AV5080 and AV5116 demonstrated better inhibitory activity against viruses bearing markers for drug resistance. Relative to their analogue counterparts, these drugs contain functional moiety modifications which may contribute to their improved inhibitory activities (Ivachtchenko et al., 2014; Ivashchenko et al., 2021). Such results indicate their potential to expand our current arsenal of influenza antivirals.

The current threshold (≥3-fold) for baloxavir susceptibility is arbitrary and more data are needed to improve laboratory data interpretation. A new cell culture-based assay, IRINA, provides improved throughput and its robustness was evident from the ability to test viruses displaying different growth kinetics. As clade 2.3.4.4b A(H5N1) viruses continue to circulate and evolve, close monitoring of their drug susceptibility is needed to inform clinical management in the event of human infections and to make informed decisions on the national stockpiling of antiviral medications.

## Supplementary Material

mmc1

mmc2

## Figures and Tables

**Table 1:** Neuraminidase inhibitor susceptibility of NGS-flagged clade 2.3.4.4b HPAI A(H5N1) viruses assessed in a neuraminidase inhibition assay.

Influenza A virus	Amino acid change in NA head^a^	Mean IC_50_ ± SD, nM (fold-change)^b^				
		Oseltamivir	Zanamivir	Peramivir	Laninamivir	AV5080

*Median IC*_*50*_ *A(H5N1)*^c^		*2.66*	*0.21*	*0.10*	*0.18*	*0.04*
chicken/ID/22	**I117T**	3.00 ± 0.37 (1)	0.29 ± 0.02 (1)	0.08 ± 0.02 (1)	0.16 ± 0.01 (1)	0.05 ± 0.01 (1)
skunk/WA/22	**I117T**	2.80 ± 0.09 (1)	0.25 ± 0.02 (1)	0.09 ± 0.00 (1)	0.15 ± 0.00 (1)	0.04 ± 0.00 (1)
fox/WI-30/22	V149I, N355S	1.30 ± 0.35 (0.5)	0.21 ± 0.01 (1)	0.09 ± 0.00 (1)	0.16 ± 0.02 (1)	0.05 ± 0.00 (1)
dolphin/FL/22	**S247N**	15.49 ± 0.45 (6)	0.32 ± 0.02 (1)	0.38 ± 0.03 (4)	0.30 ± 0.01 (2)	0.09 ± 0.01 (2)
vulture/FL-58/22	N295D	3.56 ± 0.77 (1)	0.84 ± 0.10 (4)	0.20 ± 0.05 (2)	0.39 ± 0.08 (2)	0.15 ± 0.02 (4)
vulture/FL-31/22	**N295S**, T438N	196.54 ± 30.59 (74)	18.01 ± 4.52 (86)	9.03 ± 2.22 (90)	3.36 ± 1.06 (19)	2.19 ± 0.11 (55)
vulture/FL-33/22^d^	**N/S295**, T/N438	11.05 ± 1.60 (4)	4.20 ± 0.36 (20)	0.55 ± 0.05 (6)	0.64 ± 0.10 (4)	0.14 ± 0.02 (4)
clone 1	**N295S**	37.11 ± 5.27 (14)	0.85 ± 0.15 (4)	0.59 ± 0.10 (6)	0.50 ± 0.05 (3)	0.28 ± 0.00 (7)
clone 2	T438N	4.24 ± 0.26 (2)	2.55 ± 0.25 (12)	0.18 ± 0.02 (2)	0.34 ± 0.04 (2)	0.06 ± 0.01 (2)
clone 3	**N295S**, T438N	135.15 ± 39.93 (51)	15.97 ± 0.62 (76)	7.34 ± 1.03 (73)	2.84 ± 0.28 (16)	2.03 ± 0.45 (51)
*Reference* ^e^						
IL/45/19 A (H1N1)pdm09	–	0.17 ± 0.03	0.16 ± 0.01	0.06 ± 0.01	0.20 ± 0.02	0.09 ± 0.01
AL/03/20 A (H1N1)pdm09	**H275Y**	229.67 ± 25.29 (1351)	0.22 ± 0.01 (1)	15.25 ± 2.11 (254)	0.43 ± 0.04 (2)	0.76 ± 0.07 (8)

Susceptibility of HPAI A(H5N1) viruses, grown in 10-day-old embryonated chicken eggs, was assessed using a fluorescence-based NI assay. NA inhibitors oseltamivir carboxylate, zanamivir, peramivir, and laninamivir were purchased from BioSynth, Berkshire, United Kingdom; AV5080 was provided by ChemDiv, San Diego, CA. Dash lines (−) indicate the absence of NA substitution. Antiviral testing was conducted in biosafety level 3 enhanced facility.

a Encompasses the head domain (amino acid residues 82–470) of NA (also see Supplemental Table S2 for complete virus list and information). NA amino acid substitutions previously associated with reduced drug susceptibility are shown in **bold**.

b Each virus was tested in ≥3 independent experiments to determine IC_50_ value (50% inhibitory concentration, nM); SD, standard deviation.

c A(H5N1) viruses lacking the flagged NA substitutions (n = 15) were used to determine the median IC_50_ (the baseline susceptibility). A fold change in IC_50_s of flagged A (H5N1) viruses relative to the median IC_50_ is shown.

d A/black vulture/Florida/22–012,333–001/2022 (vulture/FL-33/22) was subjected to limiting dilution procedure in MDCK-SIAT1 cells and resulting virus clones containing either a single or dual amino acid substitution at residues 295 and 438 were recovered and tested.

e From the CDC Neuraminidase Inhibitor Susceptibility Reference Virus Panel version 3.0 (International Reagent Resource, IRR: FR-1755). For reference viruses, IC_50_ fold change was determined by comparing to NA sequence-matched wildtype reference virus IC_50_.

**Table 2:** Susceptibility of NGS-flagged clade 2.3.4.4b HPAI A(H5N1) viruses to PA cap-dependent endonuclease inhibitors baloxavir and AV5116 in cell culture-based assays.

Influenza A Virus	Amino acid substitution in PA^a^	Mean EC_50_ ± SD, nM (fold-change)^b^			
		Baloxavir^c^		AV5116	
		HINT	IRINA	HINT	IRINA

*Median EC*_*50*_ *A(H5N1)*^d^		*0.57*	*0.34*	*0.52*	*0.25*
chicken/PA-6/22	**A37T**	3.21 ± 0.82 (6)	2.06 ± 0.59 (6)	3.15 ± 0.28 (6)	1.48 ± 0.16 (6)
chicken/PA-10/22	**A37T**	3.15 ± 0.74 (6)	1.84 ± 0.41 (5)	2.40 ± 0.35 (5)	1.62 ± 0.68 (6)
hawk/MN-1/22	**I38M**	3.34 ± 0.89 (6)	2.94 ± 0.62 (9)	3.12 ± 1.07 (6)	2.45 ± 0.21 (10)
chicken/MI/22	**I38T** ^e^	42.51 ± 9.40 (75)	36.84 ± 10.21 (108)	19.36 ± 4.94 (37)	19.53 ± 3.21 (78)
*Reference* ^f^					
IL/08/18 A (H1N1) pdm09	–	1.65 ± 0.44	1.12 ± 0.32	1.89 ± 0.55	1.35 ± 0.48
IL/08/18 A (H1N1) pdm09	**I38T**	128.66 ± 34.68 (78)	112.84 ± 27.26 (101)	61.87 ± 18.2 (33)	52.59 ± 10.33 (39)
LA/50/17 A (H3N2)	–	1.04 ± 0.43	0.76 ± 0.4	1.10 ± 0.37	0.81 ± 0.21
LA/49/17 A (H3N2)	**I38M**	10.58 ± 3.79 (10)	8.70 ± 3.09 (11)	12.21 ± 3.53 (11)	10.32 ± 1.94 (13)

Susceptibility to CEN inhibitors baloxavir acid (baloxavir) (Shionogi and Co., Ltd., Osaka, Japan) and AV5116 (ChemDiv, San Diego, CA) was determined in MDCK-SIAT1 cells using HINT (High-content Imaging Neutralization Test) and IRINA (Influenza Replication Inhibition Neuraminidase-based Assay). Dash lines (−) indicate the absence of PA substitution. Antiviral testing was conducted in biosafety level 3 enhanced facility.

a Encompasses the endonuclease active site (amino acid residues 1–200) of the PA protein (also see Supplemental Table S3 for complete virus list and information). PA amino acid substitutions previously associated with reduced drug susceptibility are shown in **bold**.

b Each virus was tested in ≥3 independent runs to determine EC_50_ value (50% effective concentration, nM); SD, standard deviation.

c Baloxavir acid, active metabolite form of the pro-drug baloxavir marboxil.

d A(H5N1) viruses lacking the flagged PA substitutions (n = 17) were used to determine the median EC_50_ (baseline susceptibility). Fold-change in EC_50_s of flagged A(H5N1) virus relative to the median EC_50_ is shown.

e A/chicken/Michigan/22–013,961–001-original/2022 (chicken/MI/22) contains two additional amino acid substitutions M61I and A85T within the PA endonuclease active site.

f From the CDC Baloxavir Susceptibility Reference Virus Panel version 1.1 (IRR: FR-1678). For reference viruses, EC_50_ fold-change was determined by comparing to PA sequence-matched wildtype reference virus EC_50_. An arbitrary threshold (≥3-fold) is used to report PA amino acid substitutions that confer reduced susceptibility to baloxavir.

## Data Availability

Data will be made available on request.

## References

[R1] Alkie TN, Cox S, Embury-Hyatt C, Stevens B, Pople N, Pybus MJ, Xu W, Hisanaga T, Suderman M, Koziuk J, Kruczkiewicz P, Nguyen HH, Fisher M, Lung O, Erdelyan CNG, Hochman O, Ojkic D, Yason C, Bravo-Araya M, Bourque L, Bollinger TK, Soos C, Giacinti J, Provencher J, Ogilvie S, Clark A, MacPhee R, Parsons GJ, Eaglesome H, Gilbert S, Saboraki K, Davis R, Jerao A, Ginn M, Jones MEB, Berhane Y, 2023. Characterization of neurotropic HPAI H5N1 viruses with novel genome constellations and mammalian adaptive mutations in free-living mesocarnivores in Canada. Emerg. Microb. Infect 12, 2186608. 10.1080/22221751.2023.2186608 PMC10026807 36880345

[R2] Bevins SN, Shriner SA, Cumbee JC Jr., Dilione KE, Douglass KE, Ellis JW, Killian ML, Torchetti MK, Lenoch JB, 2022. Intercontinental movement of highly pathogenic avian influenza A(H5N1) clade 2.3.4.4 virus to the United States, 2021. Emerg. Infect. Dis 28, 1006–1011. 35302933 10.3201/eid2805.220318 PMC9045435

[R3] Boltz DA, Douangngeun B, Phommachanh P, Sinthasak S, Mondry R, Obert C, Seiler P, Keating R, Suzuki Y, Hiramatsu H, Govorkova EA, Webster RG, 2010. Emergence of H5N1 avian influenza viruses with reduced sensitivity to neuraminidase inhibitors and novel reassortants in Lao People’s Democratic Republic. J. Gen. Virol 91, 949–959. 20016036 10.1099/vir.0.017459-0 PMC2888158

[R4] Centers for Disease Control and Prevention, 2022. Antiviral Drugs for Seasonal Influenza: Additional Links and Resources https://www.cdc.gov/flu/professionals/antivirals/links.htm. (Accessed 19 April 2023).

[R5] Centers for Disease Control and Prevention, 2023. Technical Report: Highly Pathogenic Avian Influenza A(H5N1) Viruses https://www.cdc.gov/flu/avianflu/spotlights/2022-2023/h5n1-technical-report.htm. (Accessed 19 April 2023).

[R6] Cox NJ, Trock SC, Burke SA, 2014. Pandemic preparedness and the influenza risk assessment tool (IRAT). Curr. Top. Microbiol. Immunol 385, 119–136. 25085014 10.1007/82_2014_419

[R7] Creanga A, Hang NLK, Cuong VD, Nguyen HT, Phuong HVM, Thanh LT, Thach NC, Hien PT, Tung N, Jang Y, Balish A, Dang NH, Duong MT, Huong NT, Hoa DN, Tho ND, Klimov A, Kapella BK, Gubareva L, Kile JC, Hien NT, Mai LQ, Davis CT, 2017. Highly pathogenic avian influenza A(H5N1) viruses at the animal-human interface in vietnam, 2003–2010. J. Infect. Dis 216, S529–S538. 28934457 10.1093/infdis/jix003

[R8] Earhart KC, Elsayed NM, Saad MD, Gubareva LV, Nayel A, Deyde VM, Abdelsattar A, Abdelghani AS, Boynton BR, Mansour MM, Essmat HM, Klimov A, Shuck-Lee D, Monteville MR, Tjaden JA, 2009. Oseltamivir resistance mutation N294S in human influenza A(H5N1) virus in Egypt. J Infect Public Health 2, 74–80. 20701864 10.1016/j.jiph.2009.04.004

[R9] Elsmo EJ, Wünschmann A, Beckmen KB, Broughton-Neiswanger LB, Buckles EL, Ellis J, Fitzgerald SD, Gerlach R, Hawkins S, Ip HS, Lankton JS, Lemley EM, Lenoch JB, Killian ML, Lantz K, Long L, Maes R, Mainenti M, Melotti J, Moriarty ME, Nakagun S, Ruden RM, Shearn-Bochsler V, Thompson D, Torchetti MK, Van Wettere AJ, Wise AG, Lim AL, 2023. Pathology of Natural Infection with Highly Pathogenic Avian Influenza Virus (H5N1) Clade 2.3.4.4b in Wild Terrestrial Mammals in the United States in 2022. bioRvix10.3201/eid2912.230464 PMC10683806 37987580

[R10] Global Influenza Programme Wep, 2020. Global Influenza Programme WEP Tool for Influenza Pandemic Risk Assessment (TIPRA) WHO (second ed.) (2020), p. 65 2nd ed.

[R11] Gubareva LV, Hayden F, 2006. M2 and neuraminidase inhibitors: anti-influenza activity, mechanism of resistance, and clinical effectiveness. In: Kawaoka Y (Ed.), Influenza Virology: Current Topics Caister Academic Press, England, 1269 – 202.

[R12] Gubareva LV, Mishin VP, Patel MC, Chesnokov A, Nguyen HT, De La Cruz J, Spencer S, Campbell AP, Sinner M, Reid H, Garten R, Katz JM, Fry AM, Barnes J, Wentworth DE, 2019. Assessing baloxavir susceptibility of influenza viruses circulating in the United States during the 2016/17 and 2017/18 seasons. Euro Surveill 24. 10.2807/1560-7917.ES.2019.24.3.1800666 PMC6344838 30670144

[R13] Ikematsu H, Kawai N, 2011. Laninamivir octanoate: a new long-acting neuraminidase inhibitor for the treatment of influenza. Expert Rev. Anti Infect. Ther 9, 851–857. 21973296 10.1586/eri.11.112

[R14] Ivachtchenko AV, Ivanenkov YA, Mitkin OD, Yamanushkin PM, Bichko VV, Shevkun NA, Karapetian RN, Leneva IA, Borisova OV, Veselov MS, 2014. Novel oral anti-influenza drug candidate AV5080. J. Antimicrob. Chemother 69, 1892–1902. 24729605 10.1093/jac/dku074

[R15] Ivashchenko AA, Mitkin OD, Jones JC, Nikitin AV, Koryakova AG, Karapetian RN, Kravchenko DV, Mochalov SV, Ryakhovskiy AA, Aladinskiy V, Leneva IA, Falynskova IN, Glubokova EA, Govorkova EA, Ivachtchenko AV, 2021. Synthesis, inhibitory activity and oral dosing formulation of AV5124, the structural analogue of influenza virus endonuclease inhibitor baloxavir. J. Antimicrob. Chemother 76, 1010–1018. 33367751 10.1093/jac/dkaa524 PMC7953317

[R16] Jones JC, Yen HL, Adams P, Armstrong K, Govorkova EA, 2023. Influenza antivirals and their role in pandemic preparedness. Antivir. Res 210, 105499. 36567025 10.1016/j.antiviral.2022.105499 PMC9852030

[R17] Kandeil A, Patton C, Jones J, Jeevan T, Harrington W, Trifkovic S, Seiler J, Fabrizio T, Woodard K, Turner J, Crumpton JC, Miller L, Rubrum A, DeBeauchamp J, Russell C, Govorkova E, Vogel P, Torchetti M, Berhane Y, Stallknecht D, Poulson R, Kercher L, Webby RJ, 2023. Rapid evolution of A (H5N1) influenza viruses after intercontinental spread to North America. Nat. Commun 14, 3082. 37248261 10.1038/s41467-023-38415-7 PMC10227026

[R18] Kode SS, Pawar SD, Tare DS, Keng SS, Hurt AC, Mullick J, 2019. A novel I117T substitution in neuraminidase of highly pathogenic avian influenza H5N1 virus conferring reduced susceptibility to oseltamivir and zanamivir. Vet. Microbiol 235, 21–24. 31282375 10.1016/j.vetmic.2019.06.005

[R19] Krammer F, Schultz-Cherry S, 2023. We need to keep an eye on avian influenza. Nat. Rev. Immunol 23, 267–268. 36944755 10.1038/s41577-023-00868-8 PMC10028763

[R20] Le MT, Wertheim HF, Nguyen HD, Taylor W, Hoang PV, Vuong CD, Nguyen HL, Nguyen HH, Nguyen TQ, Nguyen TV, Van TD, Ngoc BT, Bui TN, Nguyen BG, Nguyen LT, Luong ST, Phan PH, Pham HV, Nguyen T, Fox A, Nguyen CV, Do HQ, Crusat M, Farrar J, Nguyen HT, de Jong MD, Horby P, 2008. Influenza A H5N1 clade 2.3.4 virus with a different antiviral susceptibility profile replaced clade 1 virus in humans in northern Vietnam. PLoS One 3, e3339. 18836532 10.1371/journal.pone.0003339 PMC2556101

[R21] Le QM, Kiso M, Someya K, Sakai YT, Nguyen TH, Nguyen KH, Pham ND, Ngyen HH, Yamada S, Muramoto Y, Horimoto T, Takada A, Goto H, Suzuki T, Suzuki Y, Kawaoka Y, 2005. Avian flu: isolation of drug-resistant H5N1 virus. Nature 437, 1108. 16228009 10.1038/4371108a

[R22] Lee N, Hurt AC, 2018. Neuraminidase inhibitor resistance in influenza: a clinical perspective. Curr. Opin. Infect. Dis 31, 520–526. 30299356 10.1097/QCO.0000000000000498

[R23] McKimm-Breschkin JL, Barrett S, Wong FYK, Pudjiatmoko Azhar M, Selleck P, Davies KR, Hartaningsih N, McGrane J, 2018. Identification of Indonesian clade 2.1 highly pathogenic influenza A(H5N1) viruses with N294S and S246N neuraminidase substitutions which further reduce oseltamivir susceptibility. Antivir. Res 153, 95–100. 29574145 10.1016/j.antiviral.2018.03.007

[R24] McKimm-Breschkin JL, Selleck PW, Usman TB, Johnson MA, 2007. Reduced sensitivity of influenza A (H5N1) to oseltamivir. Emerg. Infect. Dis 13, 1354–1357. 18252107 10.3201/eid1309.07-0164 PMC2857286

[R25] Naughtin M, Dyason JC, Mardy S, Sorn S, von Itzstein M, Buchy P, 2011. Neuraminidase inhibitor sensitivity and receptor-binding specificity of Cambodian clade 1 highly pathogenic H5N1 influenza virus. Antimicrob. Agents Chemother 55, 2004–2010. 21343450 10.1128/AAC.01773-10 PMC3088255

[R26] Nguyen HT, Nguyen T, Mishin VP, Sleeman K, Balish A, Jones J, Creanga A, Marjuki H, Uyeki TM, Nguyen DH, Nguyen DT, Do HT, Klimov AI, Davis CT, Gubareva LV, 2013. Antiviral susceptibility of highly pathogenic avian influenza A(H5N1) viruses isolated from poultry, Vietnam, 2009–2011. Emerg. Infect. Dis 19, 1963–1971. 24274711 10.3201/eid1912.130705 PMC3840871

[R27] Okomo-Adhiambo M, Sleeman K, Ballenger K, Nguyen HT, Mishin VP, Sheu TG, Smagala J, Li Y, Klimov AI, Gubareva LV, 2010. Neuraminidase inhibitor susceptibility testing in human influenza viruses: a laboratory surveillance perspective. Viruses 2, 2269–2289. 21994620 10.3390/v2102269 PMC3185571

[R28] Patel MC, Flanigan D, Feng C, Chesnokov A, Nguyen HT, Elal AA, Steel J, Kondor RJ, Wentworth DE, Gubareva LV, Mishin VP, 2022. An optimized cell-based assay to assess influenza virus replication by measuring neuraminidase activity and its applications for virological surveillance. Antivir. Res 208, 105457. 36332755 10.1016/j.antiviral.2022.105457 PMC10149149

[R29] Skog E, Nykvist M, Naguib MM, Wille M, Brojer C, Agarwal V, Ellstrom P, Westman G, Lundkvist A, Jarhult JD, 2023. An oseltamivir-resistant avian H1N1 influenza A virus can transmit from mallards to chickens similarly to a wild-type strain: implications for the risk of resistance transmission to humans. J. Gen. Virol 104. 10.1099/jgv.0.001835 37018118

[R30] Sood R, Kumar N, Bhatia S, Chanu KV, Gupta CL, Pateriya AK, Mishra A, Khandia R, Mawale N, Singh VP, 2018. Neuraminidase inhibitors susceptibility profiles of highly pathogenic influenza A (H5N1) viruses isolated from avian species in India (2006–2015). Antivir. Res 158, 143–146. 30125616 10.1016/j.antiviral.2018.08.007

[R31] World Health Organization, 2023. Global Influenza Program: Laboratory Methodologies for Testing the Antiviral Susceptibility of Influenza Viruses https://www.who.int/teams/global-influenza-programme/laboratory-network/quality-assurance/antiviral-susceptibility-influenza. (Accessed 30 March 2023).

[R32] Yasuhara A, Yamayoshi S, Kiso M, Sakai-Tagawa Y, Okuda M, Kawaoka Y, 2022. A broadly protective human monoclonal antibody targeting the sialidase activity of influenza A and B virus neuraminidases. Nat. Commun 13, 6602. 36329075 10.1038/s41467-022-34521-0 PMC9632566

