# Supplementary material for: Antiviral susceptibility of clade 2.3.4.4b highly pathogenic avian influenza A(H5N1) viruses isolated from birds and mammals in the United States, 2022
Source: Antiviral Res. Author manuscript; Available in PMC 2023 Sep 19. (PMC10508830; doi:10.1016/j.antiviral.2023.105679)
Supplement: mmc1 [file NIHMS1921577-supplement-mmc1.docx]

**SUPPLEMENTARY MATERIAL**

**Viruses**

Clade 2.3.4.4b highly pathogenic avian influenza (HPAI) A(H5N1) viruses were obtained from US Department of Agriculture (USDA), Animal Plant Health Inspection Service. Virus stocks were prepared by inoculation of 10-day old embryonated chicken eggs incubated at 37°C. Allantoic fluids were harvested after 24 h, clarified by centrifugation, aliquoted, and stored at -80°C until use. Next-generation sequence (NGS) analysis identified a mixed virus population in the neuraminidase (NA) of A/black vulture/Florida/22-012333-001/2022 at residues 295 [(asparagine (N) or serine (S)] and 438 [threonine (T) or N)], present in both the isolate received and prepared virus stock. Mixed virus populations were separated by a conventional limiting dilution procedure in MDCK-SIAT1 cells using 96-well microplates. Cloned viruses were subjected to NGS analysis and three cloned viruses containing NA mutants containing N295S, T438N, or both N295S+T438N were tested in NI assay (Table S2).

CDC antiviral susceptibility reference virus panels (International Reagent Resource (IRR); FR-1755 and FR-1678) were used as controls for comparison in phenotypic assays. All virus handling and antiviral testing was conducted in biosafety level 3 enhanced laboratories.

**NGS and sequence analysis**

Codon-complete genome sequences of the panel viruses were obtained by NGS using Illumina platform and analyzed by the IRMA (iterative refinement meta-assembler) approach with single-nucleotide variant threshold of 5% (Shepard et al., 2016). The NA, PA and M2 gene sequences of clade 2.3.4.4b HPAI A(H5N1) viruses collected in the US during 2022 and deposited in Global Initiative on Sharing All Influenza Data (GISAID) database were downloaded and analyzed. The sequences were aligned using MAFFT version 7 program (Katoh & Standley, 2013).

**Antiviral compounds**

The NA inhibitors oseltamivir carboxylate (oseltamivir), zanamivir, peramivir, and laninamivir were purchased from BioSynth (Berkshire, United Kingdom) and were used to prepare stocks at concentration of 50 µM. Baloxavir acid (baloxavir) was obtained from Shionogi and Co., Ltd. (Osaka, Japan). NA inhibitor AV5080 and CEN inhibitor AV5116 were provided by ChemDiv, Inc (San Diego, CA, USA). Baloxavir, AV5080, and AV5116 were dissolved in 100% DMSO (Sigma, St. Louis, MO) to concentrations of 50 µM (AV5080) or 1 mM (baloxavir and AV5116). Antiviral stocks were stored at ≤ -30°C until use.

**Neuraminidase Inhibition assay**

NA activity and NI assays were carried out with the NA-Fluor kit (Applied Biosystems, Waltham, MA) as previously described (Okomo-Adhiambo et al., 2013). Briefly, viruses were normalized to have an NA activity that produces 10 µM of fluorescent product 4-methylumbelliferone (4-MU) upon cleavage of substrate 2-(4-(methylumbelliferyl)-a-D-N-acetylneuraminic acid (MUNANA). Normalized virus preparations were incubated in the presence of NA inhibitors, and fluorescence (Ex = 360 nm; Em = 460 nm) was measured using a Cytation 7 cell imaging multimode reader (Agilent-Biotek, Santa Clara, CA), and the IC_50_ (the drug concentration required to inhibit NA activity by 50%) was calculated. Means and standard deviations (SD) were calculated from results collected from at least three independent tests.

**High-content Imaging-based Neutralization Test (HINT) and Influenza Replication Inhibition Neuraminidase-based Assay (IRINA)**

The cell culture-based assays HINT and IRINA were performed as previously described (Jorquera et al., 2021; Patel et al 2022) with minor modifications. Briefly, MDCK-SIAT1 (0.7 × 10^5^ cells/well) were added to wells of a 96-well microplate (black clear bottom, Agilent) containing virus diluted to produce ~1000 infected cells and of serially diluted inhibitor and incubated at 37°C in 5% CO_2_ for 8-10 hrs. Diluent for virus, antiviral, and cells was DMEM supplemented with 0.2% bovine serum albumin, 25 mM HEPES, 100 U/mL penicillin, 100 ug/mL streptomycin. For HINT assay, cells were fixed and immuno-stained with a mouse anti-NP antibody followed by secondary staining with sheep anti-mouse IgG antibody conjugated to Alexa Fluor™ 555 (1:1000; Thermo Fisher Scientific, Waltham, MA) and Hoechst 33258 (1:5000; AnaSpec Inc., Fremont, CA). Fluorescence imaging and infected cell counts were done using Cytation 7. For IRINA, supernatant was aspirated, and NA activity of the cell monolayer was measured using NA Fluor-kit, where 50 µL of substrate was added to each well following incubation at 37°C in 5% CO_2_. After 1h, 50 µL stop solution was added and fluorescence was measured from the bottom of the microplate using Cytation 7. EC_50_ values were determined as previously described for HINT and IRINA by curve-fitting using nonlinear regression (Okomo-Adhiambo et al., 2013; Jorquera et al., 2019; Patel et al., 2022).

**References**

Katoh, K., Standley, D.M., 2013. MAFFT multiple sequence alignment software version 7: improvements in performance and usability. Mol Biol Evol 30, 772-780.

Jorquera, P.A., Mishin, V.P., Chesnokov, A., Nguyen, H.T., Mann, B., Garten, R., Barnes, J., Hodges, E., De La Cruz, J., Xu, X., Katz, J., Wentworth, D.E., Gubareva, L.V., 2019. Insights into the antigenic advancement of influenza A(H3N2) viruses, 2011-2018. Sci Rep 9, 2676.

Okomo-Adhiambo, M., Sleeman, K., Ballenger, K., Nguyen, H.T., Mishin, V.P., Sheu, T.G., Smagala, J., Li, Y., Klimov, A.I., Gubareva, L.V., 2010. Neuraminidase inhibitor susceptibility testing in human influenza viruses: a laboratory surveillance perspective. Viruses 2, 2269-2289.

Patel, M.C., Flanigan, D., Feng, C., Chesnokov, A., Nguyen, H.T., Elal, A.A., Steel, J., Kondor, R.J., Wentworth, D.E., Gubareva, L.V., Mishin, V.P., 2022. An optimized cell-based assay to assess influenza virus replication by measuring neuraminidase activity and its applications for virological surveillance. Antiviral Res 208, 105457.

Shepard, S.S., Meno, S., Bahl, J., Wilson, M.M., Barnes, J., Neuhaus, E., 2016. Viral deep sequencing needs an adaptive approach: IRMA, the iterative refinement meta-assembler. BMC Genomics 17, 708.

Table S1. Flagged clade 2.3.4.b HPAI A(H5N1) viruses containing amino acid substitutions previously associated with resistance or potentially decreased susceptibility to FDA-approved influenza antivirals.

| **A(H5N1) virus name** | **GISAID**  **Isolate ID**^a^ | **State** | **Host** | **Collected** | **Substitution** | |
| --- | --- | --- | --- | --- | --- | --- |
|  |  |  |  |  | **Protein** | **Position** |
| A/domestic duck/South Dakota/22-033350-004-original/2022 | EPI_ISL_16297334 | South Dakota | wild bird | 10/18/2022 | M2 | V27A |
| A/turkey/South Dakota/22-033350-005-original/2022 | EPI_ISL_16297335 | South Dakota | poultry | 10/18/2022 | M2 | V27A |
| A/chicken/South Dakota/22-033350-001-original/2022 | EPI_ISL_16297337 | South Dakota | poultry | 10/18/2022 | M2 | V27A |
| A/chicken/Idaho/22-011347-004-original/2022 | EPI_ISL_15077371 | Idaho | poultry | 4/14/2022 | NA | I117T |
| A/skunk/Washington/22-019274-001-original/2022 | EPI_ISL_15078254 | Washington | mammal | 6/7/2022 | NA | I117T |
| A/turkey/South Dakota/22-009839-001-original/2022 | EPI_ISL_13052422 | South Dakota | poultry | 3/30/2022 | NA | Q136P |
| A/duck/Washington/22-016021-001-original/2022 | EPI_ISL_16244029 | Washington | poultry | 5/22/2022 | NA | V149F |
| A/duck/Washington/22-016021-002-original/2022 | EPI_ISL_16244030 | Washington | poultry | 5/22/2022 | NA | V149F |
| A/chicken/Minnesota/22-010928-001-original/2022 | EPI_ISL_15004376 | Minnesota | poultry | 4/11/2022 | NA | V149I |
| A/turkey/Minnesota/22-010928-002-original/2022 | EPI_ISL_15004377 | Minnesota | poultry | 4/11/2022 | NA | V149I |
| A/guinea fowl/Minnesota/22-010928-003-original/2022 | EPI_ISL_15004378 | Minnesota | wild bird | 4/11/2022 | NA | V149I |
| A/domestic duck/Minnesota/22-010928-004-original/2022 | EPI_ISL_15004379 | Minnesota | wild bird | 4/11/2022 | NA | V149I |
| A/fox/Wisconsin/22-014746-021-original/2022 | EPI_ISL_17260666 | Wisconsin | mammal | 5/6/2022 | NA | V149I |
| A/fox/Wisconsin/22-014746-026-original/2022 | EPI_ISL_17260667 | Wisconsin | mammal | 5/6/2022 | NA | V149I |
| A/fox/Wisconsin/22-014746-030-original/2022 | EPI_ISL_15078247 | Wisconsin | mammal | 5/6/2022 | NA | V149I |
| A/chicken/North Dakota/22-036126-001-original/2022 | EPI_ISL_16271854 | North Dakota | poultry | 11/8/2022 | NA | V149I |
| A/domestic duck/New Jersey/22-032412-001-original/2022 | EPI_ISL_16297363 | New Jersey | wild bird | 10/11/2022 | NA | D199E |
| A/chicken/Delaware/22-006945-001-original/2022 | EPI_ISL_11897673 | Delaware | poultry | 3/7/2022 | NA | S247G |
| A/bottlenose dolphin/Florida/UFTt2203/2022^b^ | EPI_ISL_15069397 | Florida | mammal | 3/30/2022 | NA | S247N |
| A/dolphin/Florida/22-025319-002-original/2022 | EPI_ISL_15078255 | Florida | mammal | 3/30/2022 | NA | S247N |
| A/turkey/New Hampshire/22-007886-001-original/2022 | EPI_ISL_11897697 | New Hampshire | poultry | 3/15/2022 | NA | H275Y |
| A/chicken/Maine/22-008540-001-original/2022 | EPI_ISL_11971483 | Maine | poultry | 3/22/2022 | NA | H275Y |
| A/great horned owl/Massachusetts/22MM00199/2022 | EPI_ISL_16641778 | Massachusetts | wild bird | 3/3/2022 | NA | H275Y |
| A/Canada goose/Massachusetts/22-025071-002-original/2022 | EPI_ISL_16632523 | Massachusetts | wild bird | 7/21/2022 | NA | H275Y |
| A/black vulture/Florida/22-010358-001-original/2022 | EPI_ISL_15077374 | Florida | wild bird | 3/31/2022 | NA | N295D |
| A/black vulture/Florida/22-012331-001-original/2022 | EPI_ISL_15077376 | Florida | wild bird | 4/15/2022 | NA | N295S |
| A/black vulture/Florida/22-012333-001-original/2022^c^ | EPI_ISL_15077377 | Florida | wild bird | 4/16/2022 | NA | N295S |
| A/chicken/Minnesota/22-012966-001-original/2022 | EPI_ISL_16171457 | Minnesota | poultry | 4/26/2022 | PA | K34R |
| A/fox/New York/103994/2022 | EPI_ISL_14553811 | New York | mammal | 5/10/2022 | PA | A36V |
| A/chicken/Iowa/22-033362-001-original/2022 | EPI_ISL_16297336 | Iowa | poultry | 10/19/2022 | PA | A36V |
| A/chicken/Pennsylvania/22-012092-006-original/2022 | EPI_ISL_15077373 | Pennsylvania | poultry | 4/19/2022 | PA | A37T |
| A/chicken/Pennsylvania/22-012092-010-original/2022 | EPI_ISL_15078261 | Pennsylvania | poultry | 4/19/2022 | PA | A37T |
| A/chicken/Pennsylvania/22-012092-005-original/2022 | EPI_ISL_16171249 | Pennsylvania | poultry | 4/19/2022 | PA | A37T |
| A/chicken/North Dakota/22-012538-001-original/2022 | EPI_ISL_16171409 | North Dakota | poultry | 4/25/2022 | PA | A37T |
| A/Cooper's hawk/Minnesota/22-012931-001-original/2022 | EPI_ISL_15078240 | Minnesota | wild bird | 4/26/2022 | PA | I38M |
| A/chicken/Michigan/22-013961-001-original/2022 | EPI_ISL_15078244 | Michigan | poultry | 5/4/2022 | PA | I38T |
| A/harbor seal/Maine/22-020455-001-original/2022 | EPI_ISL_14098915 | Maine | mammal | 6/24/2022 | PA | I38V |
| A/harbor seal/Maine/22-020455-002-original/2022 | EPI_ISL_14098916 | Maine | mammal | 6/24/2022 | PA | I38V |
| A/harbor seal/Maine/22-020983-007-original/2022 | EPI_ISL_14098924 | Maine | mammal | 6/29/2022 | PA | I38V |

PA, NA, and M gene segments of clade 2.3.4.4b HPAI A(H5N1) viruses collected in the United States and deposited in the Global Initiative on Sharing All Influenza Data (GISAID) database (accessed on 03/23/2023).

^a^Sequences were compiled and analyzed using MAFFT version 7 program.

^b^Sequence data submitted came from the same virus as that submitted by USDA (A/dolphin/Florida/22-025319-002-original/2022: GISAID isolate ID EPI_ISL_15078255).

^c^A/black vulture/Florida/22-012333-001/2022 contained mixed virus populations at residues 295 and 438.

Table S2. Neuraminidase inhibitor susceptibility of a panel containing representative and flagged clade 2.3.4.4b HPAI A(H5N1) 97 viruses collected in US, 2022.

| **Influenza A virus** | **Abbreviation** | **USDA genotype** | **Amino acid**  **change in NA**^a^ | **Mean IC_50_ ± SD, nM (fold-change)**^b^ | | | | | **GISAID Isolate ID** |
| --- | --- | --- | --- | --- | --- | --- | --- | --- | --- |
|  |  |  |  | **Oseltamivir** | **Zanamivir** | **Peramivir** | **Laninamivir** | **AV5080** |  |
| *Median IC_50_ A(H5N1)*^c^ |  |  |  | *2.66* | *0.21* | *0.10* | *0.18* | *0.04* |  |
| A/American wigeon/South Carolina/22-000345-001/2021 | wigeon/SC/21 | A1 | D259E | 2.72 ± 0.12 (1) | 0.32 ± 0.02 (2) | 0.12 ± 0.01 (1) | 0.21 ± 0.03 (1) | NT | EPI_ISL_17008863 |
| A/bald eagle/Florida/22-006544-004/2022^d^ | eagle/FL/22 | B1.1 | - | 3.01 ± 0.74 (1) | 0.23 ± 0.03 (1) | 0.08 ± 0.01 (1) | 0.17 ± 0.02 (1) | 0.04 ± 0.01 (1) | EPI_ISL_15063846 |
| A/turkey/Iowa/22-012098-001/2022 | turkey/IA/22 | B2.1 | - | 2.66 ± 1.10 (1) | 0.20 ± 0.02 (1) | 0.10 ± 0.01 (1) | 0.18 ± 0.01 (1) | 0.04 ± 0.00 (1) | EPI_ISL_15078238 |
| A/black vulture/Maryland/22-012407-001/2022 | vulture/MD/22 | B1.1 | - | 2.31 ± 0.44 (1) | 0.21 ± 0.03 (1) | 0.10 ± 0.02 (1) | 0.18 ± 0.05 (1) | 0.05 ± 0.02 (1) | EPI_ISL_15078239 |
| A/Cooper’s hawk/Minnesota/22-012931-001/2022 | hawk/MN-1/22 | B2.1 | **-** | 2.73 ± 0.05 (1) | 0.21 ± 0.04 (1) | 0.10 ± 0.01 (1) | 0.21 ± 0.05 (1) | 0.04 ± 0.00 (1) | EPI_ISL_15078240 |
| A/red-shouldered hawk/Minnesota/22-012000-004/2022 | hawk/MN-4/22 | B2.1 | - | 2.39 ± 0.20 (1) | 0.18 ± 0.02 (1) | 0.09 ± 0.01 (1) | 0.18 ± 0.01 (1) | 0.03 ± 0.00 (1) | EPI_ISL_15077375 |
| A/fox/Wisconsin/22-013774-002/2022 | fox/WI-2/22 | B2.1 | - | 2.78 ± 0.77 (1) | 0.18 ± 0.01 (1) | 0.09 ± 0.01 (1) | 0.19 ± 0.01 (1) | 0.04 ± 0.00 (1) | EPI_ISL_13052717 |
| A/Canada goose/Wyoming/22-011671-001/2022 | goose/WY/22 | B2.1 | - | 2.70 ± 0.35 (1) | 0.18 ± 0.01 (1) | 0.08 ± 0.01 (1) | 0.16 ± 0.01 (1) | 0.03 ± 0.00 (1) | EPI_ISL_15078237 |
| A/fox/Michigan/22-014536-004/2022 | fox/MI/22 | B1.2 | del50-70^e^ | 2.55 ± 0.06 (1) | 0.21 ± 0.01 (1) | 0.10 ± 0.00 (1) | 0.17 ± 0.02 (1) | 0.04 ± 0.00 (1) | EPI_ISL_15078245 |
| A/chicken/Pennsylvania/22-012092-006/2022 | chicken/PA-6/22 | A1 | G105S | 1.64 ± 0.34 (1) | 0.21 ± 0.01 (1) | 0.11 ± 0.01 (1) | 0.20 ± 0.02 (1) | 0.06 ± 0.02 (2) | EPI_ISL_15077373 |
| A/chicken/Pennsylvania/22-012092-010/2022 | chicken/PA-10/22 | A1 | G105S | 1.91 ± 0.67 (1) | 0.25 ± 0.08 (1) | 0.14 ± 0.04 (1) | 0.21 ± 0.02 (1) | 0.08 ± 0.01 (2) | EPI_ISL_15078261 |
| A/bald eagle/Wyoming/22-013015-001/2022 | eagle/WY/22 | B4.1 | S82L, V114A | 3.22 ± 0.44 (1) | 0.30 ± 0.04 (1) | 0.15 ± 0.01 (2) | 0.25 ± 0.04 (1) | 0.06 ± 0.01 (2) | EPI_ISL_15078241 |
| A/chicken/Idaho/22-011347-004/2022 | chicken/ID/22 | B2.1 | **I117T** | 3.00 ± 0.37 (1) | 0.29 ± 0.02 (1) | 0.08 ± 0.02 (1) | 0.16 ± 0.01 (1) | 0.05 ± 0.01 (1) | EPI_ISL_15077371 |
| A/skunk/Washington/22-019274-001/2022 | skunk/WA/22 | B3.2 | **I117T** | 2.80 ± 0.09 (1) | 0.25 ± 0.02 (1) | 0.09 ± 0.00 (1) | 0.15 ± 0.00 (1) | 0.04 ± 0.00 (1) | EPI_ISL_15078254 |
| A/fox/Wisconsin/22-014746-030/2022 | fox/WI-30/22 | B3.1 | V149I, N355S | 1.30 ± 0.35 (0.5) | 0.21 ± 0.01 (1) | 0.09 ± 0.00 (1) | 0.16 ± 0.02 (1) | 0.05 ± 0.00 (1) | EPI_ISL_15078247 |
| A/Virginia opossum/Iowa/22-016780-001/2022 | opossum/IA/22 | B1.2 | I188T | 2.98 ± 0.44 (1) | 0.21 ± 0.01 (1) | 0.09 ± 0.02 (1) | 0.16 ± 0.02 (1) | 0.05 ± 0.00 (1) | EPI_ISL_15078250 |
| A/dolphin/Florida/22-025319-002/2022 | dolphin/FL-2/22 | B1.2 | **S247N** | 15.49 ± 0.45 (6) | 0.32 ± 0.02 (1) | 0.38 ± 0.03 (4) | 0.30 ± 0.01 (2) | 0.09 ± 0.01 (2) | EPI_ISL_15078255 |
| A/black vulture/Florida/22-010358-001/2022 | vulture/FL-58/22 | B1.1 | N295D | 3.56 ± 0.77 (1) | 0.84 ± 0.10 (4) | 0.20 ± 0.05 (2) | 0.39 ± 0.08 (2) | 0.15 ± 0.02 (4) | EPI_ISL_15077374 |
| A/black vulture/Florida/22-012331-001/2022 | vulture/FL-31/22 | B1.1 | **N295S**, T438N | 196.54 ± 30.59 (74) | 18.01 ± 4.52 (86) | 9.03 ± 2.22 (90) | 3.36 ± 1.06 (19) | 2.19 ± 0.11 (55) | EPI_ISL_15077376 |
| A/black vulture/Florida/22-012333-001/2022 | vulture/FL-33/22^f^ | B1.1 | **N/S295**, T/N438 | 11.05 ± 1.60 (4) | 4.20 ± 0.36 (20) | 0.55 ± 0.05 (6) | 0.64 ± 0.10 (4) | 0.14 ± 0.02 (4) | EPI_ISL_15077377 |
| A/black vulture/Florida/22-012333-001/2022 clone1 | clone 1 | B1.1 | **N295S** | 37.11 ± 5.27 (14) | 0.85 ± 0.15 (4) | 0.59 ± 0.10 (6) | 0.50 ± 0.05 (3) | 0.28 ± 0.00 (7) | EPI_ISL_18039387 |
| A/black vulture/Florida/22-012333-001/2022 clone2 | clone 2 | B1.1 | T438N | 4.24 ± 0.26 (2) | 2.55 ± 0.25 (12) | 0.18 ± 0.02 (2) | 0.34 ± 0.04 (2) | 0.06 ± 0.01 (2) | EPI_ISL_18039385 |
| A/black vulture/Florida/22-012333-001/2022 clone3 | clone 3 | B1.1 | **N295S**, T438N | 135.15 ± 39.93 (51) | 15.97 ± 0.62 (76) | 7.34 ± 1.03 (73) | 2.84 ± 0.28 (16) | 2.03 ± 0.45 (51) | EPI_ISL_18039386 |
| A/harbor seal/Maine/22-020983-002/2022 | seal/ME-2/22 | A2 | T362I | 2.26 ± 0.27 (1) | 0.18 ± 0.01 (1) | 0.09 ± 0.01 (1) | 0.14 ± 0.02 (1) | 0.04 ± 0.00 (1) | EPI_ISL_14098921 |
| A/chicken/Michigan/22-013961-001/2022 | chicken/MI/22 | B1.2 | I443T | 2.48 ± 0.41 (1) | 0.21 ± 0.01 (1) | 0.09 ± 0.01 (1) | 0.16 ± 0.01 (1) | 0.04 ± 0.00 (1) | EPI_ISL_15078244 |
| *Reference*^g^ |  |  |  |  |  |  |  |  |  |
| A/Illinois/45/2019 A(H1N1)pdm09 | IL/45/19 | N/A | - | 0.17 ± 0.03 | 0.16 ± 0.01 | 0.06 ± 0.01 | 0.2 ± 0.02 | 0.09 ± 0.01 | EPI_ISL_462743 |
| A/Alabama/03/2020 (A(H1N1)pdm09 | AL/03/20 | N/A | **H275Y** | 229.67 ± 25.29 (1351) | 0.22 ± 0.01 (1) | 15.25 ± 2.11 (254) | 0.43 ± 0.04 (2) | 0.76 ± 0.07 (8) | EPI_ISL_462737 |

Susceptibility of HPAI A(H5N1) viruses, grown in 10-day-old embryonated chicken eggs (clones 1-3 were grown in MDCK-SIAT1 cells), was assessed using a fluorescence-based NI assay. Dash lines (-) indicate the absence of NA substitution. NT, not tested. Pending, sequences were deposited in GISAID but isolate IDs are not yet available.

^a^NA amino acid substitutions previously associated with reduced drug susceptibility are shown in **bold**.

^b^Each virus was tested in ≥3 independent experiments to determine IC_50_ value (50% inhibitory concentration, nM); SD, standard deviation.

^c^A(H5N1) viruses lacking the flagged NA substitutions (n = 15) were used to determine the median IC_50_ (the baseline susceptibility). A fold change in IC_50_s of flagged A(H5N1) viruses relative to the median IC_50_ is shown.

^d^Sequence data was submitted under the name A/bald eagle/Florida/W22-134-OP/2022 in GISAID.

^e^A/fox/Michigan/22-014536-004/2022 (fox/MI/22) contains a 20-amino acid deletion in its NA stalk region (amino acid position 50 - 70).

^f^A/black vulture/Florida/22-012333-001/2022 (vulture/FL-33/22) was cloned by limiting dilution procedure and resulting virus clones containing a single or dual amino acid substitution at residues 295 and 438 were recovered and tested.

^g^CDC Neuraminidase Inhibitor Susceptibility Reference Virus Panel version 3.0 (IRR: FR-1755). IC_50_ fold change was determined using IC_50_ of wildtype reference virus.

Table S3. Susceptibility of a panel containing representative and flagged HPAI A(H5N1) viruses to PA cap-dependent endonuclease inhibitors baloxavir and AV5116.

| **Influenza A virus** | **Abbreviation** | **USDA Genotype** | **Amino acid substitution in PA**^a^ | **Mean EC_50_ ± SD, nM (fold-change)**^b^ | | | | **GISAID Isolate ID** |
| --- | --- | --- | --- | --- | --- | --- | --- | --- |
|  |  |  |  | **Baloxavir**^c^ | | **AV5116** | |  |
|  |  |  |  | **HINT** | **IRINA** | **HINT** | **IRINA** |  |
| *Median EC_50_ A(H5N1)*^d^ |  |  |  | *0.57* | *0.34* | *0.52* | *0.25* |  |
| A/American wigeon/South Carolina/22-000345-001/2021 | wigeon/SC/21 | A1 | - | 0.40 ± 0.11 (1) | 0.18 ± 0.06 (1) | NT | NT | EPI_ISL_17008863 |
| A/turkey/Iowa/22-012098-001/2022 | turkey/IA/22 | B2.1 | - | 0.41 ± 0.08 (1) | 0.22 ± 0.08 (1) | 0.35 ± 0.07 (1) | 0.22 ± 0.14 (1) | EPI_ISL_15078238 |
| A/bald eagle/Florida/22-006544-004/2022^e^ | eagle/FL/22 | B1.1 | - | 0.63 ± 0.16 (1) | 0.41 ± 0.10 (1) | 0.49 ± 0.12 (1) | 0.21 ± 0.08 (1) | EPI_ISL_15063846 |
| A/black vulture/Florida/22-012331-001/2022 | vulture/FL-31/22 | B1.1 | - | 0.63 ± 0.12 (1) | 0.44 ± 0.04 (1) | 0.52 ± 0.13 (1) | 0.37 ± 0.12 (1) | EPI_ISL_15077376 |
| A/black vulture/Florida/22-010358-001/2022 | vulture/FL-58/22 | B1.1 | - | 0.62 ± 0.17 (1) | 0.33 ± 0.10 (1) | 0.57 ± 0.18 (1) | 0.22 ± 0.02 (1) | EPI_ISL_15077374 |
| A/black vulture/Maryland/22-012407-001/2022 | vulture/MD/22 | B1.1 | - | 0.50 ± 0.11 (1) | 0.28 ± 0.05 (1) | 0.48 ± 0.10 (1) | 0.29 ± 0.08 (1) | EPI_ISL_15078239 |
| A/skunk/Washington/22-019274-001/2022 | skunk/WA/22 | B3.2 | - | 0.46 ± 0.11 (1) | 0.26 ± 0.05 (1) | 0.37 ± 0.08 (1) | 0.23 ± 0.04 (1) | EPI_ISL_15078254 |
| A/fox/Wisconsin/22-013774-002/2022 | fox/WI-2/22 | B2.1 | - | 0.43 ± 0.05 (1) | 0.23 ± 0.05 (1) | 0.41 ± 0.06 (1) | 0.25 ± 0.11 (1) | EPI_ISL_13052717 |
| A/fox/Wisconsin/22-014746-030/2022 | fox/WI-30/22 | B3.1 | - | 0.71 ± 0.18 (1) | 0.32 ± 0.11 (1) | 0.74 ± 0.25 (1) | 0.25 ± 0.05 (1) | EPI_ISL_15078247 |
| A/Canada goose/Wyoming/22-011671-001/2022 | goose/WY/22 | B2.1 | - | 0.47 ± 0.08 (1) | 0.29 ± 0.10 (1) | 0.40 ± 0.17 (1) | 0.17 ± 0.05 (1) | EPI_ISL_15078237 |
| A/red-shouldered hawk/Minnesota/22-012000-004/2022 | hawk/MN-4/22 | B2.1 | K29R | 0.53 ± 0.12 (1) | 0.35 ± 0.16 (1) | 0.47 ± 0.18 (1) | 0.24 ± 0.10 (1) | EPI_ISL_15077375 |
| A/chicken/Pennsylvania/22-012092-006/2022 | chicken/PA-6/22 | A1 | **A37T** | 3.21 ± 0.82 (6) | 2.06 ± 0.59 (6) | 3.15 ± 0.28 (6) | 1.48 ± 0.16 (6) | EPI_ISL_15077373 |
| A/chicken/Pennsylvania/22-012092-010/2022 | chicken/PA-10/22 | A1 | **A37T** | 3.15 ± 0.74 (6) | 1.84 ± 0.41 (5) | 2.40 ± 0.35 (5) | 1.62 ± 0.68 (6) | EPI_ISL_15078261 |
| A/Cooper’s hawk/Minnesota/22-012931-001/2022 | hawk/MN-1/22 | B2.1 | **I38M** | 3.34 ± 0.89 (6) | 2.94 ± 0.62 (9) | 3.12 ± 1.07 (6) | 2.45 ± 0.21 (10) | EPI_ISL_15078240 |
| A/chicken/Michigan/22-013961-001/2022 | chicken/MI/22 | B1.2 | **I38T,** M61I, A85T | 42.51 ± 9.40 (75) | 36.84 ± 10.21 (108) | 19.36 ± 4.94 (37) | 19.53 ± 3.21 (78) | EPI_ISL_15078244 |
| A/bald eagle/Wyoming/22-013015-001/2022 | eagle/WY/22 | B4.1 | V44A | 0.86 ± 0.14 (2) | 0.37 ± 0.08 (1) | 0.67 ± 0.13 (1) | 0.35 ± 0.08 (1) | EPI_ISL_15078241 |
| A/Virginia opossum/Iowa/22-016780-001/2022 | opossum/IA/22 | B1.2 | M61I, A85T | 0.92 ± 0.12 (2) | 0.43 ± 0.18 (1) | 0.79 ± 0.23 (2) | 0.57 ± 0.16 (2) | EPI_ISL_15078250 |
| A/fox/Michigan/22-014536-004/2022 | fox/MI/22 | B1.2 | M61I, A85T | 0.80 ± 0.13 (1) | 0.45 ± 0.06 (1) | 0.67 ± 0.14 (1) | 0.40 ± 0.06 (2) | EPI_ISL_15078245 |
| A/dolphin/Florida/22-025319-002/2022 | dolphin/FL-2/22 | B1.2 | M61I, A85T | 0.61 ± 0.06 (1) | 0.40 ± 0.12 (1) | 0.57 ± 0.07 (1) | 0.24 ± 0.07 (1) | EPI_ISL_15078255 |
| A/harbor seal/Maine/22-020983-002/2022 | seal/ME-2/22 | A2 | A70V | 0.72 ± 0.06 (1) | 0.49 ± 0.14 (1) | 0.55 ± 0.12 (1) | 0.36 ± 0.07 (1) | EPI_ISL_14098921 |
| A/chicken/Idaho/22-011347-004/2022 | chicken/ID/22 | B2.1 | I129T, I171M | 0.43 ± 0.10 (1) | 0.21 ± 0.06 (1) | 0.39 ± 0.15 (1) | 0.20 ± 0.11 (1) | EPI_ISL_15077371 |
| A/black vulture/Florida/22-012333-001/2022 | vulture/FL-33/22 | B1.1 | R185G | 0.52 ± 0.20 (1) | 0.35 ± 0.22 (1) | 0.58 ± 0.27 (1) | 0.32 ± 0.15 (1) | EPI_ISL_15077377 |
| *Reference*^f^ |  |  |  |  |  |  |  |  |
| A/Illinois/08/2018 A(H1N1)pdm09 | IL/08/18 | N/A | - | 1.65 ± 0.44 | 1.12 ± 0.32 | 1.89 ± 0.55 | 1.35 ± 0.48 | EPI_ISL_315855 |
| A/Illinois/08/2018 A(H1N1)pdm09 | IL/08/18 | N/A | **I38T** | 128.66 ± 34.68 (78) | 112.84 ± 27.26 (101) | 61.87 ± 18.2 (33) | 52.59 ± 10.33 (39) | EPI_ISL_348120 |
| A/Louisiana/50/2017 A(H3N2) | LA/50/17 | N/A | - | 1.04 ± 0.43 | 0.76 ± 0.40 | 1.10 ± 0.37 | 0.81 ± 0.21 | EPI_ISL_315857 |
| A/Louisiana/49/2017 (A(H3N2) | LA/49/17 | N/A | **I38M** | 10.58 ± 3.79 (10) | 8.70 ± 3.09 (11) | 12.21 ± 3.53 (11) | 10.32 ± 1.94 (13) | EPI_ISL_315858 |

Susceptibility to CEN inhibitors baloxavir acid (Shionogi and Co., Ltd., Osaka, Japan) and AV5116 (ChemDiv, San Diego, CA) was determined in MDCK-SIAT1 cells using HINT (High-content Imaging Neutralization Test) and IRINA (Influenza Replication Inhibition Neuraminidase-based Assay). NT, not tested.

^a^Encompasses the endonuclease active site (amino acid residues 1- 200) of the PA protein. Flagged PA amino acid substitutions associated with reduced drug susceptibility are shown in **bold**_._ Dash lines (-) indicate the absence of substitution in this site.

^b^Each virus was tested in ≥3 independent runs to determine mean and standard deviation EC_50_ value (50% effective concentration, nM).

^c^Baloxavir acid, active metabolite form of baloxavir marboxil.

^d^A(H5N1) viruses lacking the flagged PA substitutions (n = 17) were used to determine the median EC_50_ (baseline susceptibility). Fold-change in EC_50_s of A(H5N1) viruses relative to the median EC_50_ (baseline) is shown.

^e^Sequence data was submitted under the name A/bald eagle/Florida/W22-134-OP/2022 in GISAID.

^f^CDC Baloxavir Susceptibility Reference Virus Panel version 1.1 (IRR: FR-1678). EC_50_ fold-change of reference viruses was determined by comparing EC_50_ of PA sequence-matched wildtype reference virus. An arbitrary threshold (≥ 3-fold) is used to report PA amino acid substitutions that confer reduced susceptibility to baloxavir.Table S4. Effect of the dual NA substitution N295S+T438N on enzyme activity of clade 2.3.4.4B A(H5N1) viruses.

| **Influenza A(H5N1) virus** | **USDA Genotype** | **Amino acid change in NA** | **Hemagglutination titer (HAU)** | | **Infectivity (log_10_TCID_50_)** | **NA activity^a^** | | | | | |  |
| --- | --- | --- | --- | --- | --- | --- | --- | --- | --- | --- | --- | --- |
|  |  |  | **tRBC** | **gpRBC** |  | **RFU/HAU (tRBC)** | **%^b^** | **RFU/HAU (gpRBC)** | **%^b^** | **RFU/**  **TCID_50_)** | **%^b^** | |
| A/black vulture/Maryland/22-012407-001/2022 | B1.1 | - | 128 | 256 | 8.45 | 2720 | 100 | 1445 | 100 | 0.0227 | 100 | |
| A/black vulture/Florida/22-012333-001/2022 clone1 | B1.1 | N295S | 16 | 64 | 7.45 | 1761 | 64.7 | 445 | 30.8 | 0.017 | 74.9 | |
| A/black vulture/Florida/22-012333-001/2022 clone2 | B1.1 | T438N | 64 | 192 | 8.45 | 593 | 21.8 | 212 | 14.7 | 0.0023 | 10.1 | |
| A/black vulture/Florida/22-012333-001/2022 clone3 | B1.1 | N295S+T438N | 64 | 128 | 7.95 | 16 | 0.6 | 6 | 0.4 | 0.0002 | 0.9 | |

Viruses were grown in MDCK-SIAT1 cells for 48 hrs in the absence of TPCK-trypsin. Hemagglutination titers (HAU) were obtained following standard hemagglutination assay and using 0.5% turkey (tRBC) or 0.75% guinea pig red blood cells (gpRBC). Virus infectious titers were determined in MDCK-SIAT1 cells and expressed as log_10_ 50% tissue culture infectious dose (TCID_50_).

^a^NA enzyme activity of viruses was measured using the NA-Fluor kit with MUNANA substrate and expressed as relative fluorescence unit (RFU) per HAU or TCID_50_. Values shown are average from two replicates.

^b^Wildtype virus, A/black vulture/Maryland/22-012407-001/2022, was used as control to calculate the effect of NA substitutions on enzyme activity. NA amino acid sequence of A/black vulture/Maryland/22-012407-001/2022 is identical to those of the clones of A/black vulture/Florida/22-012333-001/2022, except for the substitutions shown.

**Supplementary Figure Legend**

Spread of clade 2.3.4.4b HPAI A(H5N1) virus in MDCK-SIAT1 cells at 10 h post-infection. Egg-grown HPAI A(H5N1) viruses were diluted to produce ~1000 infected cells. Single-cell suspension of MDCK-SIAT1 cells (0.7 × 10^5^ cells/well) were added into each well containing diluted virus. Plates were incubated for 10 h at 37°C with 5% CO_2_. After fixation, cells were immuno-stained and images were captured using Cytation 7. Genotypes (assigned by USDA) for each virus is shown.
