# Supplementary material for: Antiviral susceptibility of clade 2.3.4.4b highly pathogenic avian influenza A(H5N1) viruses isolated from birds and mammals in the United States, 2022
Source: Antiviral Res. Author manuscript; Available in PMC 2023 Sep 19. (PMC10508830; doi:10.1016/j.antiviral.2023.105679)
Supplement: mmc2 [file NIHMS1921577-supplement-mmc2.pptx]

## Slide 1
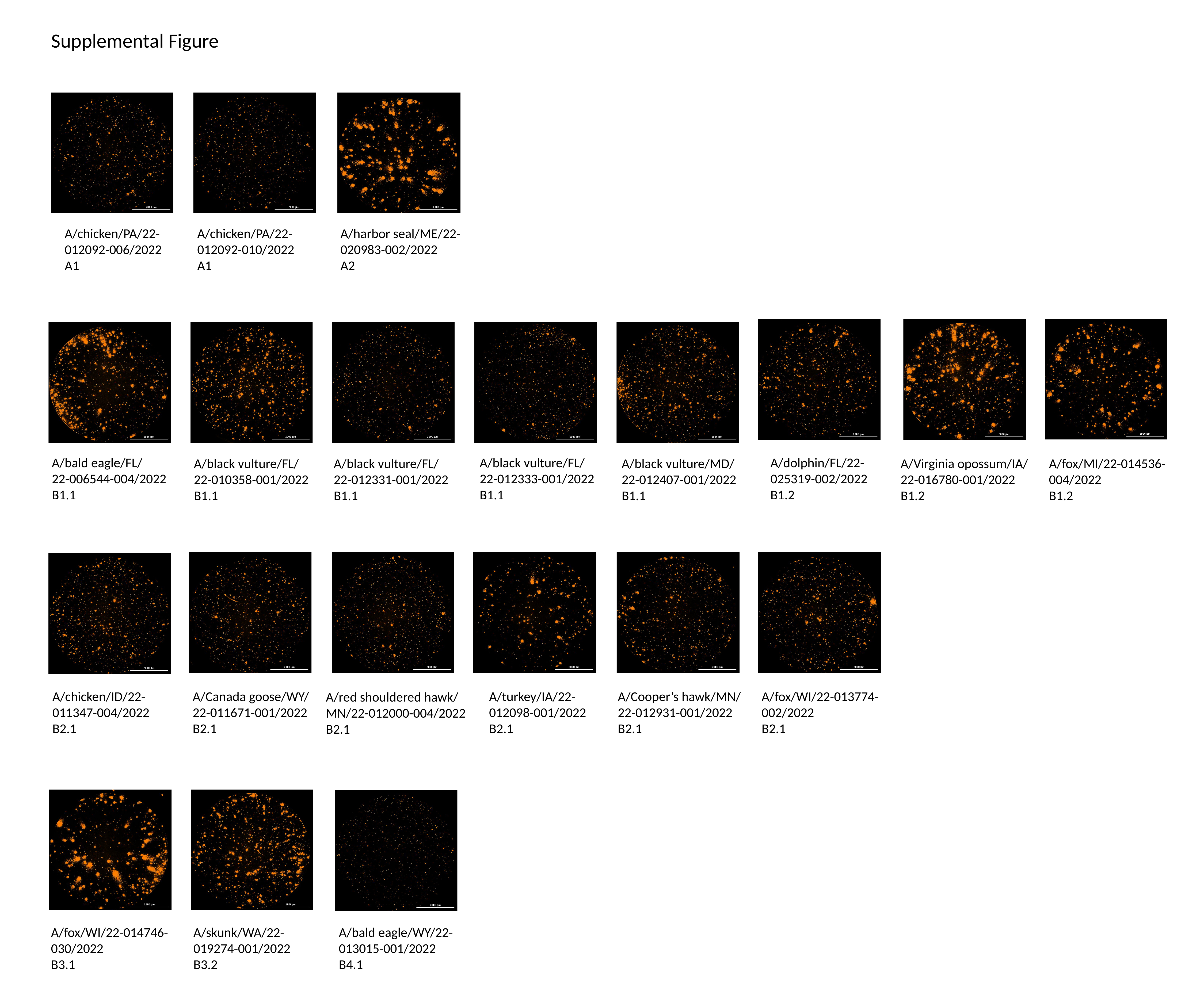

Supplemental Figure
A/chicken/PA/22-012092-006/2022
A1
A/harbor seal/ME/22-020983-002/2022
A2
A/chicken/PA/22-012092-010/2022
A1
A/bald eagle/FL/ 22-006544-004/2022
B1.1
A/black vulture/FL/ 22-012333-001/2022
B1.1
A/dolphin/FL/22-025319-002/2022
B1.2
A/Virginia opossum/IA/ 22-016780-001/2022
B1.2
A/fox/MI/22-014536-004/2022
B1.2
A/black vulture/FL/ 22-010358-001/2022
B1.1
A/black vulture/FL/ 22-012331-001/2022
B1.1
A/black vulture/MD/ 22-012407-001/2022
B1.1
A/fox/WI/22-013774-002/2022
B2.1
A/Cooper’s hawk/MN/ 22-012931-001/2022
B2.1
A/turkey/IA/22-012098-001/2022
B2.1
A/chicken/ID/22-011347-004/2022
B2.1
A/Canada goose/WY/ 22-011671-001/2022
B2.1
A/red shouldered hawk/ MN/22-012000-004/2022
B2.1
A/fox/WI/22-014746-030/2022
B3.1
A/skunk/WA/22-019274-001/2022 B3.2
A/bald eagle/WY/22-013015-001/2022
B4.1
